# Making complex interventions work in low resource settings: developing and applying a design focused implementation approach to deliver mental health through primary care in India

**DOI:** 10.1186/s13033-018-0181-7

**Published:** 2018-01-22

**Authors:** Rohit Ramaswamy, Rahul Shidhaye, Sharmishtha Nanda

**Affiliations:** 1 Public Health Leadership and Maternal and Child Health, University of North Carolina, Chapel Hill, USA; 2 Public Health Foundation of India, New Delhi, India; 3 CAPHRI (Care and Public Health Research Institute), Maastricht University, Maastricht, Netherlands

**Keywords:** Mental health, Primary care, Implementation science, Research, India

## Abstract

**Background:**

Globally, there is a large treatment gap for people with mental disorders, and this gap is especially extreme in Low and Middle Income Countries. This gap can be potentially bridged by integrating evidenced based mental health interventions into primary care, but there is little knowledge about how to do this well, especially in countries with weak health systems. Research into the best implementation approaches is a priority, but in order to do so, it is first necessary to adapt implementation science principles and tools for mental health services in low resource settings.

**Results:**

The frameworks that have been used to implement evidence-based behavioral health and health care interventions in High Income Countries do not directly apply to contexts where resources and processes for service delivery and support do not exist. We propose an implementation approach for low resource settings, called design-focused implementation, emphasizing the design of delivery systems using systematic design methods as precursor to implementation in severely resource constrained environments. This approach draws from existing literature in design thinking, quality implementation, improvement science and evaluation and we describe its use in creating the processes, organizations and the enabling environment for integration of mental health service delivery into primary care in India.

**Conclusions:**

Design-focused implementation will be useful for guiding research and practice in closing the implementation gap for a wide variety of complex interventions in low resource settings.

## Background and context

The first and second Lancet series on Global Mental Health [1, 2], World Health Organization’s (WHO) mhGAP Intervention Guidelines [3] and mhGAP intervention guide, version 2.0 [4], a series of papers published in PLoS Medicine [5], WHO-WONCA report on mental health in primary health care [6] and the Disease Control Priorities’ mental health volume [7], summarize the strong evidence base for integration of mental health services in primary care in Low and Middle Income Countries (LMIC). Unfortunately there is a huge gap between what is known about the effectiveness of a range of pharmacological, psychological, and social interventions that can transform lives and enhance communities and what is provided to and experienced by individuals in primary care and community care settings [8]. There is little evidence that evidence-based mental health treatments are either adopted or successfully implemented in primary care either in High Income Countries (HICs) [9] or in LMICs [10]. This is result of poor knowledge translation between those who generate evidence and those who are expected to use and apply it [11].

Our limited understanding of how best to deliver evidence-based interventions across the full range of existing health systems and in the wide diversity of possible settings, serves as the major barrier to translate evidence into practice. The Institute Of Medicine report has proposed a five-step framework to bring evidence-based interventions into practice [12]. The first three steps involve evidence generation and synthesis activities such as efficacy and effectiveness research and the development of clinical guidelines. This has been the primary focus of mental health research over the years, and has resulted in the creation of guidelines such as WHO’s mhGAP for implementing evidence-based mental health interventions in LMICs. The last two steps of the IOM framework emphasize the creation of quality metrics to assist in the improvement of the quality of care and the development of methods for implementation in practice. This is the focus of the work described in this paper.

In this paper, we present the approach used by the PRIME (*PR*ogramme for *I*mproving *M*ental Health Car*E*) project in India to develop an implementation strategy to deliver mental health interventions through the district level primary health care system. PRIME, a research program consortium funded by UK aid aims to generate evidence on the implementation and scaling up of integration of mental health services in primary care in Ethiopia, India, Nepal, South Africa and Uganda, ultimately to improve health, social and economic outcomes for individuals with depression, alcohol use disorder and psychosis [13]. In India, PRIME is implemented in the state of Madhya Pradesh through a three-way partnership involving the Ministry of Health, Government of Madhya Pradesh, Sangath (a Goa based NGO working in the sector of public mental health) and the Public Health Foundation of India. The details of the setting, baseline situational analysis focusing on broader policy environment and overall program context for implementation are described elsewhere [14].

### Implementation challenges in PRIME

PRIME project began as an implementation research effort to understand the best strategies for integrating WHO recommended mental health interventions into the Indian primary health care system. The situation analysis performed for PRIME and described elsewhere [14] brought to light the weakness of the health system in Madhya Pradesh. The infrastructure needed to deliver mental health services through the primary health care system in our project area of Sehore district, which covers an area of more than 2500 square miles and a population of 1.3 million was primarily non-existent, and even where something was in place, was non-functional. The entire district has four psychiatric beds in the general hospital (Sehore district hospital), one psychiatrist and one psychologist and no other trained mental health professional. The state of Madhya Pradesh, where Sehore is located, has no mental health policy or mental health care plan, and there was no possibility of availing of additional government resources to implement the mental health program.

### Need to design context-specific implementation approach

The situational analysis revealed more than ever the need for the adoption of a systematic implementation approach. While there has been significant work in identifying commonly used implementation strategies for improving adherence and sustainability of clinical programs and practice in the US [15], there is no analogous work in low resource settings. However, as we began reviewing the existing implementation science literature for commonly used models and frameworks that could apply to the Indian context, we found this to be a challenging endeavor. As described by Nilsen [16], the last decade has witnessed a significant increase in theories, frameworks and models, but many of them provide limited support for how they could be used, especially in low resource settings. As part of our scan of existing frameworks, we reviewed both what Nilsen refers to as “process” and “determinant” frameworks. For process frameworks, we reviewed the Quality Implementation Framework developed by Meyer and his colleagues [17] and the Interactive Systems Framework (ISF) developed by Wandersman [18]. For determinant frameworks, we included National Implementation Research Network’s (NIRN) Active Implementation Frameworks [19], Damschroder’s Consolidated Framework for Implementation Research (CFIR) [20] and the UK Medical Research Council’s (MRC) framework for implementing complex interventions [21].

Based on the review of these existing frameworks, we reached the conclusion that some common themes of these frameworks were of particular relevance to PRIME. For example, it was apparent that an adaptation of the WHO solutions to fit the Indian setting was going to be needed [22]. Developing the appropriate support systems (coaching and technical assistance) to build implementation capacity [18] was a critical need. And since there was no prior experience with implementation, the implementation approach would need process data to regularly assess implementation fidelity and quality and use this data to improve using iterative improvement methods such as the Plan-Do-Study-Act (PDSA) cycle [23].

At the same time, it was also apparent that none of the frameworks were directly applicable to settings such as Madhya Pradesh, where, as evidenced by the results of the situational analysis, the systems in place are not strong enough to provide basic capacity for implementation, and where resources are not available for intensive technical assistance and support. The activities recommended by NIRN at each implementation stage [19] or key components of quality implementation described in the Quality Implementation Framework [17] (e.g. the development of an implementation team and implementation plans, fostering a supportive organizational climate, providing training and technical assistance, collaboration between experts and practitioners and good evaluation) reinforce the idea, and rightly so, that successful implementation requires a systematic planning and build out process, and that a new program cannot simply be added on to existing operations without the appropriate supports and additional resources. But to accommodate these supports and resources, the existing system needs to have basic capability, with stable and defined service delivery processes, reliable staff, committed leadership and functioning supply chains and monitoring systems. This was not the case in the facilities where the PRIME interventions were to take place. Our implementation of a new mental health program needed to take place in a setting where the need for new services is overwhelming but where implementation readiness falls well below the lowest possible rating available in assessment instruments such as NIRN’s Hexagon tool [24]. Given the complexity and uncertainty of our environment, a formative iterative learning approach based on design thinking and the developmental evaluation concepts proposed by Patton [25] was needed since there was no pre-existing evidence that would inform an a priori selection of appropriate implementation strategies.

In addition to non-existent facility capacity, the project also had limited resources to support the implementation. The PRIME team in India had four staff members and no implementation resources within the health system, making it impossible for multi-level implementation teams and intermediary experts to guide the implementation. We also found that using the implementation assessment and planning tools recommended in the implementation science literature require a level of sophistication on the part of both users and facilitators, and was well beyond the linguistic and educational capability of many front line health workers.

As a result of these constraints, we needed to develop an implementation approach that was informed by the frameworks that were reviewed but was adapted to fit the conditions in India. Our approach focused first on designing the service delivery and support system that could be used to deliver mental health services through the existing primary healthcare facilities and then optimizing it using simplified implementation and improvement science tools. We believe that this approach, which we call “design focused implementation” can be used to implement other complex health interventions in weak health systems.

### Design focused implementation: an overview

**Fig. 1:**
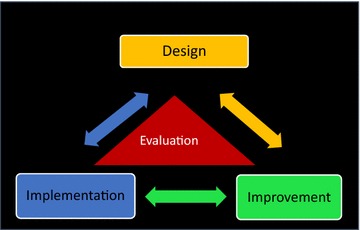
Design focused implementation model

The design focused implementation model, shown in Fig. 1, consists of three interlinked phases: *design, implementation, and improvement*. Evaluation is woven into each of these with appropriate evaluation questions for each component of the model.

We use the word “phases” in a broad sense. This is because these cannot be used in a formulaic way. They provide guidance for systematic implementation in settings where there is the need to do what is possible with limited resources, but the particular tools that are used will vary by context. *Design* refers to the development of a blueprint for implementation which involves a description of the implementation logic, an enumeration of the components or activities needed for implementation, the description, on paper, of the key processes through which service needs to be delivered, and the enabling environment needed to assure high quality delivery. To create this blueprint, principles of design thinking, defined as “*an approach to innovation that is centered on user needs and creates solutions by rapidly generating and testing new ideas*” [26] were used, particularly the idea that a deep understanding of the context of users of the service is a pre-requisite for good design, and that solutions need to be co-created with those using and operating the system [27]. Design was the driving force behind this implementation approach, and provided the guidance for the rest of the phases of the model. A viable and feasible design of the delivery system and its key components, arrived at in collaboration with users, service providers and other stakeholders was seen to be a pre-requisite for successful implementation. *Implementation* refers to the resources, capacities and support structure to execute the design and to ensure that the service delivery processes are instantiated within the health system. We use the word “implementation” here as a combination of multiple implementation stages [19], involving both pre-implementation planning activities based on the design (identification of staffing needs, training and capacity building plans, creation of operating procedures etc.) and the activities needed to deliver service, monitor performance and make iterative corrections to the design. This is because, in a system with no prior experience or capacity to deliver a new program, preparing for implementation is inextricably intertwined with the act of implementation, as the delivery processes, training materials and operating procedures were implemented and iteratively refined until they “work” in the local context. Initial assessments of readiness or prior planning were meaningless because the system was profoundly unprepared and unable to mobilize for any kind of change. Our approach therefore was to implement the design as systematically as possible, realizing that the initial implementation would be incomplete, and perhaps ineffective, and to conduct iterative modifications of the implementation until a working solution is obtained. The end of the implementation phase was the creation of a functional delivery system based on the principles of the design, but with the local adaptations needed to make it independently capable of delivering the services required by the program. The *Improvement* phase used improvement science methods to identify post-implementation performance gaps, to optimize the performance of the service delivery processes and to create a monitoring system to track performance and to proactively identify potential points of vulnerability and risk. Improvement principles were used to support implementation in two ways. Rapid improvement PDSA cycles [28] were conducted in the implementation phase for local adaptation of the design. Formal Quality Improvement (QI) project*s* using the Lean Six Sigma approach [28] were undertaken in the improvement phase to close performance gaps.

## Results

### Applying design focused implementation to PRIME: a case study

We applied the four phases of our model to implement a program for treatment of three mental disorders (depression, alcohol use disorder and psychosis) delivered across three levels of the health system (community, primary care and district hospitals). The outputs of each phase are shown in Table 1.

**Table 1:** Outputs for each phase of design focused implementation for PRIME

Design focused implementation phase	Outputs
Design	Theory of change
	Mental Health Care Plan
	Service delivery process maps
Implementation	Capacity building package
	Implementation support tools
	PRIME model customized to local context
Improvement	Reduction in PRIME model performance gaps

#### Design phase: creating a District Mental Health Care Plan

There were three outputs from the design phase: (*a*) *Theory of Change model;* (*b*) *Mental Health Care Plan* (*MHCP*) *and* (*c*) *Service Delivery Process Maps*. The situational analysis served as our starting point for the design process. Data from this analysis led to the development of a local Theory of Change (ToC) which provided the conceptual model for how to bring about change to achieve our desired outcome, given our understanding of the local situation. The ToC illustrates the pathway of linked outcomes that need to be achieved in order to successfully provide mental health care through the primary health care system in Sehore district. The ToC was developed in conjunction with key stakeholders in the government health care system representing the needs and perspectives of the service providers who are responsible for delivering the mental health program through the clinics and hospitals. The details of the ToC approach are described elsewhere [29].

**Fig. 2:**
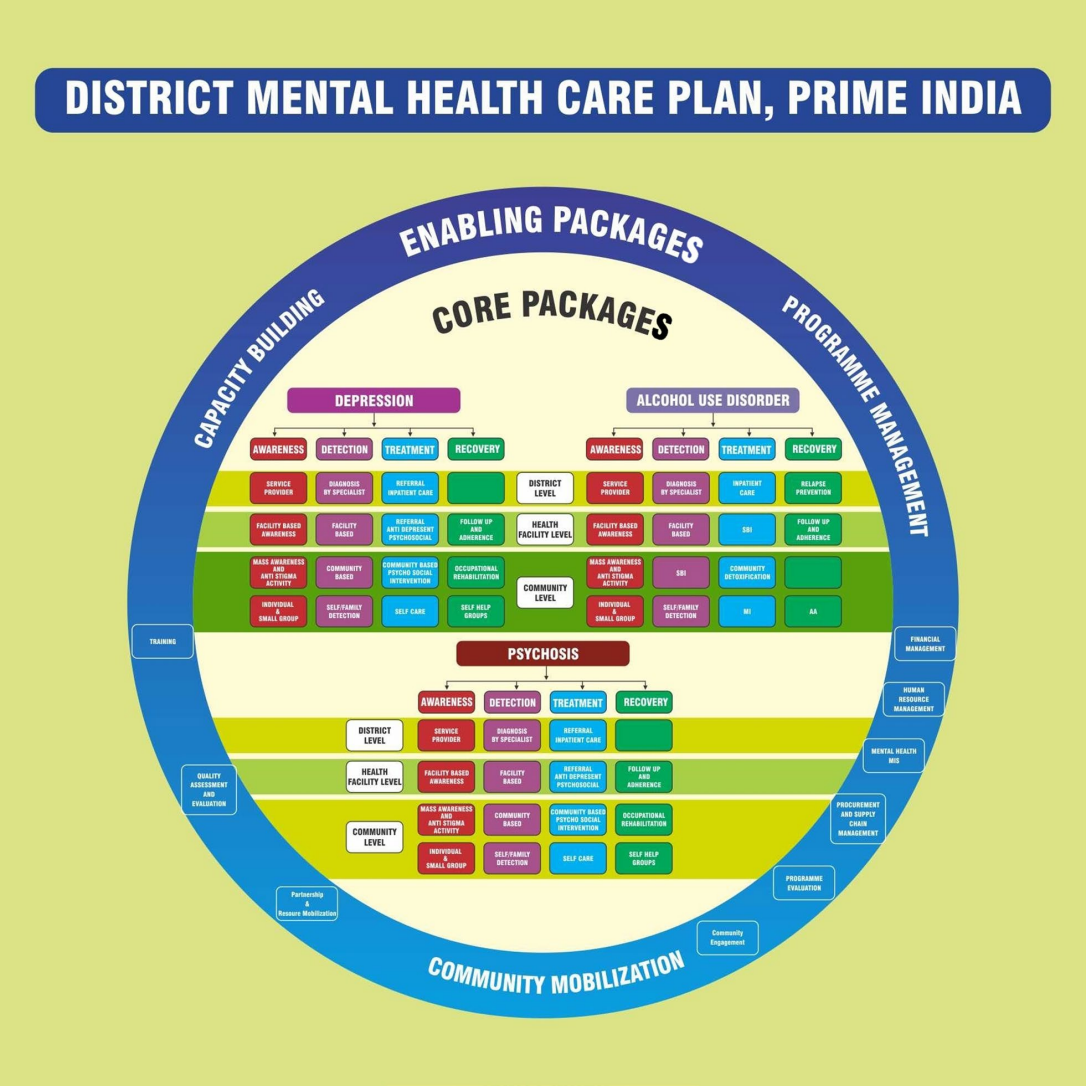
PRIME India District Mental Health Care Plan

The ToC map led to the development of the service delivery processes through which care would be delivered, as well as the systems strengthening processes needed to build system capability to support mental health delivery. The four delivery processes are *awareness, detection, treatment and recovery* for depression, alcohol use disorder and psychosis across the community, primary care and district hospitals levels. The systems strengthening processes included Health Management Information Systems (HMIS), human resources, supply chain, advocacy and monitoring and evaluation. These processes were assembled into a district *Mental Health Care Plan* (MHCP) shown in Fig. 2. The MHCP, described elsewhere in detail [30] served as the blueprint for implementation. The MHCP also served as a communication tool to reinforce to stakeholders that the program cannot be sustained without the support processes in place.

**Fig. 3:**
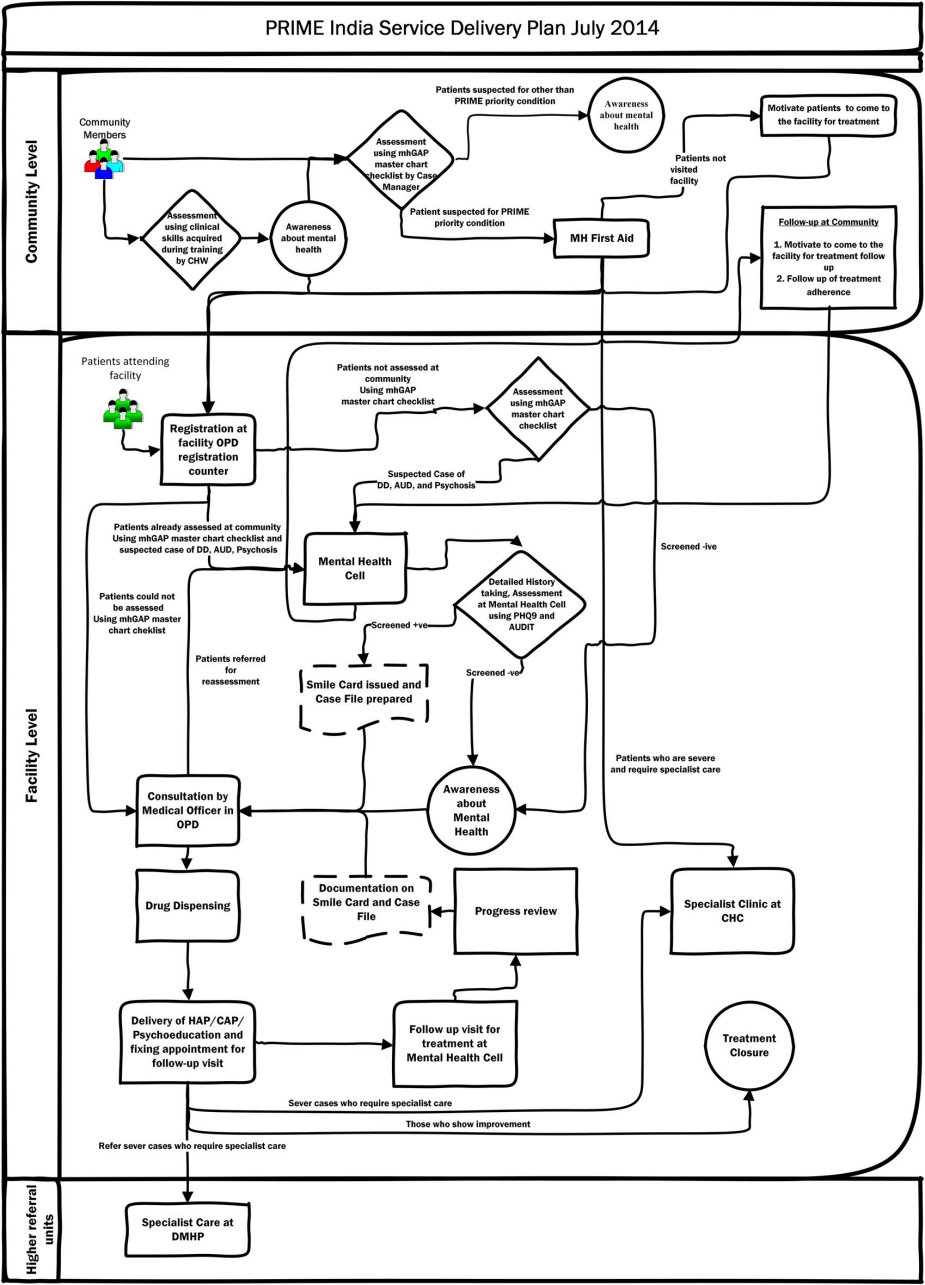
Process map for PRIME implementation

Based on the MHCP, process maps were created for the overall program implementation and for each service delivery and support process depicting the detailed flow or sequence of events that needed to take place for care to be delivered for each condition and system level. These maps used a format referred to as “cross functional mapping” that shows not only the process steps but also the organizations or people responsible for carrying out the steps, helping in the documentation of organizational hand-offs that identify potential areas of risk or poor performance affecting patient experience and outcomes. The high level process map for the entire program implementation is shown in Fig. 3.

Taken together, the ToC, the MHCP and the process maps engaged key stakeholders in the design of the various clinical and system components that needed to be in place in advance to achieve successful mental health outcomes in the PRIME setting. These outputs served to guide the activities of the implementation phase.

#### Implementation phase: developing delivery capacity and support systems

The concept for the implementation phase was adapted from the Interactive System Framework (ISF) developed by Wandersman [18], which emphasizes the need for a *support system* where capacities are built for implementation. Two types of capacities are identified in the ISF: (1) *innovation*-*specific capacity*, which is the necessary knowledge that is required for implementing and using a particular innovation and (2) *general capacity*, which represents the infrastructure, skills and motivation required to support implementation of an innovation. To build these two types of capacity, Wandersman and his colleagues identify four critical components: tools, training, technical assistance (coaching and support) and quality assurance [31].

Following this guidance, we developed three outputs from the implementation phase: (*a*) *capacity building package* (*b*) *implementation support tools and* (*c*) *PRIME service delivery model* that adapted the Mental Health Care Plan (MHCP) developed in the design phase for Sehore district. This was first implemented in one sub-district hospital. Since there was no prior experience with either the delivery system or on the best mechanisms for building capacity, an iterative approach was followed, testing adaptations of the MHCP based on data from the situational analysis and improving them through successive cycles of change based on monitoring results as described below. Retrospectively, we discovered this approach aligned with several implementation strategies enumerated in Powell et al. [15].

**Fig. 4:**
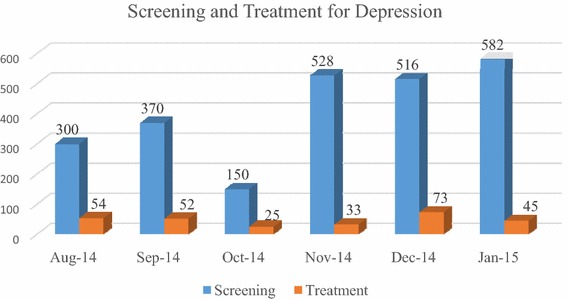
Progression of screening and treatment for depression

**Fig. 5:**
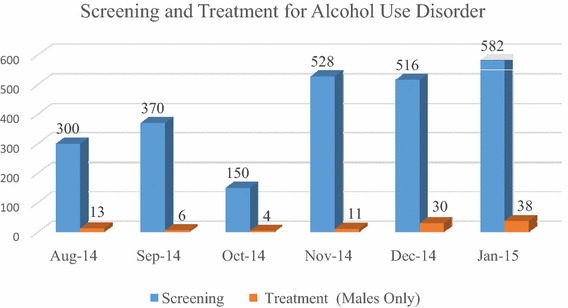
Progression of screening and treatment for alcohol use disorder

- Iteration 1: This iteration involved developing the first version of the *tools* (checklists, job aids and operating procedures based on the process maps provided to case managers and medical officers) for screening patients, provision of pharmacological and psycho-social interventions, follow-up of patients, procurement and supply chain management of drugs and establishing an information system to monitor progress of various program components. A 2 days *training* program was offered to medical officers using WHO training materials and a separate 2 days program focusing on both innovation specific capacity (detection of mental health disorders in the community) and general capacity (overview of mental health and stigma reduction) was provided to front-line workers (community health workers) and nurses. *Technical assistance* was provided through weekly face-to-face meetings with both medical officers and front-line staff with the PRIME project team and *quality assurance* was performed using a monitoring system that tracked the number of patients detected, treated and referred.
- *Iteration 2:* Eight weeks after implementation, monitoring data found poor performance on key indicators, unavailability of psychotropic drugs and lack of reporting of mental health indicators in the HMIS system. Based on this data and on interviews with the staff, it became apparent that the linkage between the community and the facility could not be sustained without intense facilitation by PRIME staff. This brought about some improvements, but this model lacked fidelity to the process design because the health system functions were now taken over by program staff. In achieving the balance between fidelity and fit of implementation of an intervention, Castro [32] emphasizes the need for invariance in the core components of a program, and this iteration violated the core design.
- *Iteration 3:* The implementation strategy for this was based on the learning from iteration 2 but tested a solution that was being promoted by the government in the new District Mental Health Program [33] and for which government resources were potentially available, but which had never been implemented. This involved the recruitment of a new resource called a “Case Manager” who provided screening functions and basic counseling services but also coordinated care within the medical officers. Case managers were also provided with tools and training and weekly support meetings, and this iteration resulted in a significant increase in detection and treatment. More details about the implementation of PRIME are provided elsewhere [30]. Figures 4 and 5 show the progress in detection and treatment of patients with depression and alcohol use disorders across the multiple iterations of the model.

#### Improvement phase: optimizing performance

While on-going improvement should be a component of any program implementation, it is especially important in weak health systems where post-implementation sustainability is an issue. The improvement phase in PRIME used a customized version of Lean Six Sigma, a popular industry-derived quality improvement (QI) approach. Six Sigma is an organized and systematic method for strategic process improvements that relies on statistical methods to make reductions in errors/defects while Lean focusses on reducing waste associated with a particular process [28]. This approach was used to identify gaps in performance post-implementation, and to bring the performance of key processes to their desired level specified by the Theory of Change. Project staff members were trained in the use of these methods, and the monitoring system used to provide data for the implementation phase iterations was also used post-implementation. After a few months of post-implementation monitoring, performance gaps were identified in two key areas: (a) the accuracy of clinical diagnosis by medical officers in the primary care facilities and (b) the rate of return of patients for follow-up counseling visits. Quality improvement projects were launched to address these issues, involving analyses to identify root causes and development of improvement solutions in consultation with case managers. Our approach in this phase is not different from standard practice, as tools in healthcare have been well documented [34]. The point we want to emphasize is the need to integrate improvement science methods such as PDSA and Lean Six Sigma into the implementation process to support and sustain the quality of implementation.

#### Evaluation phase: using learning to improve implementation

As shown in Fig. 1, evaluation activities were incorporated into each phase of the design-focused implementation approach. The specific evaluation methods used varied by phase, but were all intended to provide rapid feedback for learning and improvement, and therefore were embedded into the design, implementation and improvement phases. Our approach was informed by the principles of learning evaluation [26], which is an approach that collects real-time data to learn about and improve the implementation process. Table 2 shows how these principles were applied to evaluate the various phases of the PRIME implementation model.

**Table 2:** Application of learning evaluation principles to PRIME

Learning evaluation principles	Ways to assess principle	PRIME evaluation activity
*Principle 1* Gather data to describe types of changes made by healthcare organizations, how changes are implemented, and the evolution of the change process	Interview with healthcare organizations to establish detailed understanding of the plan for implementing change at baseline by engaging organizational leaders	Theory of change workshops to gain inputs and consensus about how the PRIME model will be implemented
	Use mixed methods to monitor how this plan evolves	Use of monitoring, observational and focus group data to track Mental Health Care Plan execution strategy
*Principle 2* Collect process and outcome data that are relevant to healthcare organizations and to the research team	Track performance on selected measures at regular time intervals throughout implementation	Monitoring system to track patients diagnosed, treated and referred throughout the implementation
*Principle 3* Assess multi-level contextual factors that affect implementation, process, outcome, and transportability	Collect qualitative and quantitative contextual data in real time	Situational analysis to understand the local service delivery context and barriers in Sehore district
	Conduct rigorous analysis to identify key contextual factors affecting outcomes	Case study to understand how the contextual factors affected the quality of implementation
*Principle 4* Assist healthcare organizations in applying data to monitor the change process and make further improvements	Assist organizations in learning from their own data to refine their innovations with a focus on continuous learning	Data used for iterative improvements to the PRIME model and for process improvement
*Principle 5* Operationalize common measurement and assessment strategies with the aim of generating transportable results	To conduct internally valid cross-organization mixed methods analyses	Common outcome indicators across the five countries of the PRIME consortium enabling cross-country comparison of outcomes and implementation strategies

The quality of the implementation process was assessed during each phase and modifications were continually made to improve implementation fidelity. During the design phase, the Theory of Change model was evaluated using two qualitative workshops conducted with state and district health officials, service providers and front line staff. Data on model accuracy was collected through individual interviews and group discussions with these stakeholders, and this data was used to modify and finalize the model and to develop the MHCP.

During the implementation phase, a mixed methods approach was used to collect evaluation data to guide the iterative cycles of change. Quantitative monitoring data was collected on the number of patients referred, the number of patients diagnosed, the number of patients treated by primary care physicians and by specialists. In addition, observational data was used to assess the quality of care and in-depth interviews and focus group discussions provided data on the acceptability and sustainability of the implementation strategy. This data was used to make iterative modifications to the execution strategy of the MHCP as described previously. Once the implementation model was finalized and stabilized across all selected facilities in the district, a field based monitoring system on the Mobenzi m-health platform was put in place to collect ongoing data on referrals, diagnoses and treatment, which served to identify improvement projects for the improvement phase. The improvement phase used statistical process control charts [35] which is standard approach to evaluate the effect of improvement interventions by collecting and plotting data regularly over time. Finally, a summative evaluation will be conducted for the entire implementation process of PRIME using a case study methodology with the objective of providing generalizable knowledge on how best to use the tools and methods of design focused implementation for future applications.

In addition, PRIME has an outcome evaluation design that uses repeated community-based cross-sectional surveys to measure change in treatment coverage, a repeated facility based survey to assess the impact on detection of disorders and disorder-specific cohorts to assess the effect of care on patient outcomes. The details of this design, which has harmonized outcome measures across the five countries of the PRIME consortium to enable cross-country comparison are described elsewhere [36], since it is not the focus of this paper.

## Discussion

In this paper, we show how different implementation science frameworks in the literature were adapted for our setting where the systems for delivery did not just need to be strengthened and supported to introduce new services, but where the core and enabling processes for implementing mental health services first needed to be designed and then integrated into the existing system. We believe that this implementation approach, and the tools presented here will be a model that practitioners implementing other programs in LMICs under similar circumstances can adopt and enhance, and will also provide important research questions about the most effective methods for successful and sustainable service delivery of programs through government health systems in low resource settings.

The need for advancements in implementation research for implementing complex interventions in low resource settings is now well established. There has been increasing attention on research to reduce the evidence-practice gap, starting from Donabedian’s classic model from half a century ago [37]. Depending on the orientation of the researchers, different terms have been used to describe these efforts including dissemination and implementation, quality assurance, quality improvement, knowledge translation, knowledge utilisation, knowledge transfer and exchange, innovation diffusion, implementation research, research utilisation, evidence-informed policy, and evidence-informed health systems [38], resulting in overlapping and confusing language that makes it difficult for practitioners to determine the best strategies needed to address their particular implementation problems. But there is general agreement that more research is needed on the tools and approaches required to implement evidence-based interventions with fidelity and quality in particular context.

Mental health is a prime example of a field that would benefit from such research, since implementing mental health programs require a combination of highly context dependent bio-medical, psycho-social and structural interventions, and there is a large translation gap from evidence to practice. However, implementation research can only be conducted in the context of implementation practice, and there is limited knowledge of the tools and approaches to embed mental health programs in weak health systems. Moreover, implementation tools and methods are scattered across a variety of disciplines such as engineering, management and psychology that are not traditionally associated with public health, and therefore are not often available in an integrated manner to support high quality implementation. Several frameworks for applied implementation exist, but they are difficult to apply in settings where service delivery and enabling processes have to be first constructed before service can be provided.

Our newly developed implementation approach, called design focused implementation, is informed by well-known implementation frameworks but was created for low resource settings where the system is weak, and resources for intensive coaching and support are unavailable. We demonstrate the application of this approach to mental health service delivery in one district in India through the use of specific tools that were relevant to this context; other tools may be added or substituted to this framework for other programs. In other PRIME settings in Ethiopia, Nepal, South Africa and Uganda, the local contextual factors guided the implementation process. In none of these settings, any formal implementation science framework was used though in practice all the research teams followed quite a similar approach to ensure implementation of their respective MHCPs. We are currently analyzing the process data from all five settings and plan to publish case studies related to implementation of MHCPs in these settings in 2018.

Conceptually, our approach provides a way of ensuring implementation quality through its entire lifecycle in low resource settings—from the creation of service delivery and systems strengthening processes, to their operationalization in a real-world setting, through ongoing improvement and optimization of performance. Using this approach systematically will result in tested implementation packages for complex interventions that can include a detailed description of the processes, organizations, technologies, supply chains, equipment, facilities etc. that are needed to put the evidence to work in practice, as well as the system performance requirements to ensure that implementation failure does not get in the way of achieving outcomes.

## Conclusion

Design-focused implementation will be useful for guiding research and practice in closing the implementation gap for mental disorders as well as a wide variety of complex interventions in low resource settings.
